# Continued Posttrial Benefits of Buprenorphine Extended Release: RECOVER Study Findings

**DOI:** 10.1097/ADM.0000000000001070

**Published:** 2022-09-16

**Authors:** Brent Boyett, Vijay R. Nadipelli, Caitlyn T. Solem, Howard Chilcoat, Warren K. Bickel, Walter Ling

**Affiliations:** From the Pathway Healthcare, Birmingham, AL (BB); Indivior, Inc, Richmond, VA (VRN, HC); Johns Hopkins Bloomberg School of Public Health, Baltimore, MD (HC); OPEN Health, Bethesda, MD (CTS); Fralin Biomedical Research Institute, Virginia Tech Carilion, Roanoke, VA (WKB); and Department of Family Medicine, David Geffen School of Medicine, University of California, Los Angeles, Los Angeles, CA (WL).

**Keywords:** opioid use disorder, treatment, buprenorphine, quality of life, symptoms, abstinence

## Abstract

This analysis describes participants’ opioid use disorder (OUD) outcomes for 18 months after discontinuing extended-release buprenorphine injection (BUP-XR, SUBLOCADE).

**Methods:**

The RECOVER (Remission From Chronic Opioid Use: Studying Environmental and Socioeconomic Factors on Recovery) study recruited participants from BUP-XR clinical trials (NCT02357901, NCT025100142, and NCT02896296) to assess whether there were sustained benefits after leaving the trial. Abstinence from opioids and from all illicit substances (excluding medical cannabis), health-related quality of life, depression, and employment were measured after BUP-XR discontinuation and change in outcomes assessed at 6, 12, and 18 months. Results were analyzed within the full cohort and by duration of BUP-XR treatment (0–2 months, 3–5 months, 6–11 months, 12 months, or 13–18 months) with and without inverse probability weights adjusting for differences in baseline characteristics.

**Results:**

Of 533 participants, 529 were assessed over the 18-month study period. Further posttrial pharmacotherapy was reported by 33% of participants. At RECOVER baseline, longer BUP-XR was associated with higher abstinence (0–2 months BUP-XR [n = 116]: 38.8%; 3–5 months BUP-XR [n = 61]: 41.0%; 6–11 months BUP-XR [n = 86]: 68.6%; 12 months BUP-XR [n = 135]: 71.9%; 18 months BUP-XR [n = 131]: 88.2%) and greater 12-Item Short Form Health Survey mental component scores. Over 60% of participants had stable or improved outcomes at 6, 12, and 18 months assessments. Overall 47% of participants self-reported sustained opioid abstinence for the full 18-month follow-up, with greater sustained abstinence associated with longer BUP-XR treatment duration. A sensitivity analysis, removing patients receiving medications for OUD, yielded similar results.

**Conclusions:**

Participants from BUP-XR clinical trials who continued into RECOVER maintained or improved on numerous outcomes over 18 months, demonstrating the long-term positive impact of OUD pharmacotherapy.

Opioid use disorder (OUD) remains a serious health crisis in the United States, with 50,042 overdose deaths in 2019^1^ and an estimated $504 billion cost attributable to the opioid crisis in 2015.^2,3^ The National Institutes of Health Helping to End Addiction Long-term initiative describes a number of areas of opportunity, which can be addressed to better understand this crisis and prioritize research initiatives,^4^ including developing new treatment strategies to improve treatment compliance, prevent relapse, reduce risk of misuse, and determine the optimal length of medication treatment for opioid addiction.

As new injectable treatments come to market, understanding the long-term outcomes for patients who have received treatment is important. These include the person’s environment and life experience, in addition to abstinence alone.^5^

The RECOVER observational study (Remission From Chronic Opioid Use: Studying Environmental and Socioeconomic Factors on Recovery; NCT03604861)^6^ provides an opportunity to better understand the long-term outcomes in persons who seek treatment with moderate to severe OUD after taking part in a trial for SUBLOCADE (hereafter, BUP-XR). The RECOVER study provides prospective data not only on abstinence but also other patient-reported outcomes that provide a more comprehensive picture of how patients experience recovery after exiting a clinical trial setting. Previous analyses of the 12-month RECOVER outcomes found that participants had positive health-related quality of life (HRQoL), minimal depression, and higher employment compared with their status before BUP-XR trials.^7^

The objectives of this analysis were to (1) describe real-world outcomes in a cohort of people with OUD over 18 months after discontinuation of BUP-XR and (2) examine whether outcomes differed among participants based on BUP-XR treatment duration.

## METHODS

### Study Design

**Figure 1:**
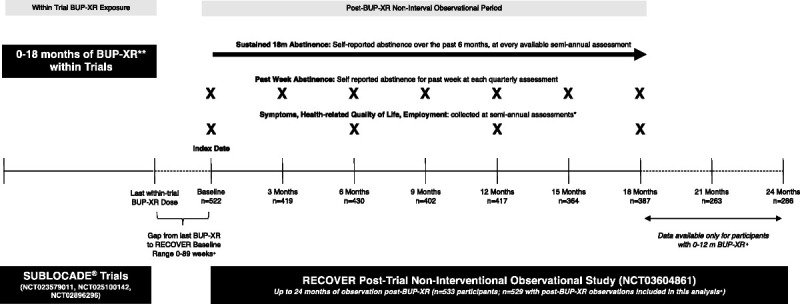
Overview of RECOVER data collection period and study sample size by visit. *Employment also captured at quarterly assessments, but only semiannual assessments included in this analysis. Opioid craving scale added as the study was ongoing and only consistently captured for 12- and 18-month assessments. +For a subgroup of participants (n = 142), the RECOVER study overlapped with a BUP-XR extension study, and as such patients with overlap were reindexed to first semiannual visit after BUP-XR discontinuation. Sample sizes per visit reflect this reindexed cohort. **BUP-XR received within trials was SUBLOCADE (Indivior, Richmond, VA).

RECOVER is an observational study assessing life changes in patients with OUD receiving up to 18 monthly BUP-XR injections as part of either a separate randomized placebo-controlled clinical efficacy trial (NCT02357901)^8,9^ and/or separate open-label safety (NCT02510014) and extension (NCT02896296) studies. RECOVER followed participants for up to 24 months from baseline after exiting BUP-XR trials (Fig. 1).

The enrollment period from the last monthly BUP-XR injection to baseline ranged from 0 to 89 weeks because many patients had already left the BUP-XR trials when RECOVER started enrolling. Also, due to the open-label extension study and RECOVER running simultaneously, some patients continued to receive injections during the first 6 months of the RECOVER baseline.

Although all participants in RECOVER could have up to 24 months of follow-up, those who were in the long-term safety study (NCT02896296) could have only up to 18 months of follow-up after their last BUP-XR injection. This analysis reindexed participants who had concurrent BUP-XR use with RECOVER, shifting visits back by 6 months, including data for the first 18 months of follow-up, to allow for the same time frame of comparison for those who were and those who were not reindexed.

Short surveys administered via in-person computer-assisted interviews and urine drug screens (UDSs) were completed every 3 months, with a more complete surveys administered every 6 months for up to 24 months. Participants who missed an assessment were still eligible to complete future assessments. RECOVER was conducted in accordance with all applicable ethical and regulatory requirements and approved by the institutional review board. Informed consent was obtained from all study participants before study participation.

Total BUP-XR treatment duration received in the period before reindexed baseline was classified as 0–2 months BUP-XR (including those receiving only placebo or 1–2 BUP-XR injections), 3–5 months BUP-XR, 6–11 months BUP-XR, 12 months BUP-XR, and 13–18 months BUP-XR. These categories were consistent with previous analyses,^7^ except for separating 12 months and 13–18 months BUP-XR treatment durations.

### Substance Use Disorder Treatment During RECOVER

Although RECOVER was a noninterventional observational study, participants could have obtained treatment for opioid and other drug use after leaving the clinical trial program. Information regarding treatment was collected during the study, including pharmacotherapy. This information was based on participant self-report.

To understand the impact of BUP-XR alone, independent of subsequent treatment, a sensitivity analysis was performed removing any patients receiving medications for opioid use disorder (MOUDs) during the follow-up period (ie, self-reported at visits 6 through 18). Because of the small number of patients receiving 13–18 months BUP-XR who did not receive further MOUD (n = 2), the 12 months and 13–18 months categories were collapsed for this sensitivity analysis.

### Outcomes

#### Abstinence From Opioids and Illicit Substances

Opioid abstinence–related outcomes were collected quarterly and included self-reported past week abstinence and UDS results.^10^ Primary analyses were based on self-report. Sensitivity analyses around opioid abstinence definitions included abstinence based on UDS results alone and a composite of self-report and UDS results. Sustained abstinence, measured as self-reported abstinence to opioids at all measured semi-annual visits, was also calculated. Self-reported use of alcohol to intoxication, cannabis (excluding medical cannabis), cocaine, methamphetamine or stimulant misuse, depressants, and hallucinogenics were reported; a composite variable for abstinence from all illicit substances excluding medical cannabis or misuse of prescription medications based on self-report was also calculated.

#### Health-related Quality of Life

Overall HRQoL was measured using the 12-Item Short Form Health Survey (SF-12), measuring patient-reported outcomes along 8 dimensions reflecting quality of life, calculating a mental component summary (MCS) and physical component summary (PCS) score.^11^ These scores reflect US population norms in terms of physical and mental health with a mean score of 50 and a standard deviation (SD) of 10 points.

In addition, the Beck Depression Inventory II (BDI-II) was included to assess depression within this cohort. The BDI-II is a 21-item self-report rating inventory measuring depression symptoms and severity.^12–14^ This scale ranges from 0 to 63, with higher scores indicating greater depression.^14^ For our study, the question regarding suicide was excluded from the measure due to the sensitive nature of the question.

#### Employment

Employment status (ie, employed full- or part-time) was self-reported quarterly by the participant.

### Statistical Analysis

Our primary hypothesis was that patients who have been treated with BUP-XR would continue to experience stable or improved outcomes over time. A secondary hypothesis was that the duration of BUP-XR received is associated with more positive outcomes (ie, more patients with stable positive outcomes or improved outcomes). Outcomes at RECOVER baseline for the full cohort and by BUP-XR treatment duration group were described, assessing whether scores improved, remained stable, or declined compared with their values at baseline using χ^2^ tests.

For dichotomous outcomes (ie, abstinence, employment), improvement was defined as changing from the negative state to the positive (eg, from nonabstinence to abstinence), stability was defined as no change from the score at RECOVER baseline and was stratified by whether outcomes remained positive or negative, and decline was defined as a switch from the positive to negative state (Table 1). For continuous or ordinal scales, decline was defined as a negative change in scores that was greater than the minimal clinically important difference (MCID). Although a difference of approximately 1.5–3 points has been established as MCID based on different methodologies,^12,13^ there is a lack of empirical support for this definition.^12^ In the absence of a clear MCID threshold, we used an empirically defined threshold of 0.5 times the baseline SD for each measure (thus, 4 for SF-12 PCS, 6 for SF-12 MCS, and 5 for BDI-II); stability was defined as a change that was less than an MCID in either a positive or negative direction; and improvement was defined as a positive change that was greater than the MCID (Table 1).

**TABLE 1:** Definitions for Improvement, Decline, and Stability From Baseline in Each Outcome

	Decline	Stability	Improvement
Abstinence outcomes	Abstinent participants move to nonabstinence	Abstinent participants remain abstinent (stable-abstinent); nonabstinent participants remain non abstinent (stable-nonabstinent)	Nonabstinent participants move to being abstinent
Depression	Increase in BDI-II of >5 points	Improvement or decline in BDI-II of ≤5 points	Decrease in BDI-II of >5 points
Health-related quality of life	Decrease in PCS of SF-12 of >4 points Decrease in MCS of SF-12 of >6 points	Improvement or decline in PCS of SF-12 of ≤4 points Improvement or decline in MCS of SF-12 of ≤6 points	Increase in PCS of SF-12 of >4 points Increase in MCS of SF-12 of >6 points
Employment	Employed participants move to unemployed	Employed participants remain employed (stable-employed); Unemployed participants remain unemployed (stable-unemployed)	Unemployed participants move to being employed

BDI-II, Brief Depression Index II; MCS, Mental Component Summary; PCS, Physical Component Summary; SF-12, 12-Item Short Form Health Survey.

Because patients were not randomized to receive different durations of BUP-XR within trials but these durations were driven by study length and potentially patient characteristics in BUP-XR trials, comparisons by BUP-XR treatment duration group were conducted both before and after applying inverse probability weights. These weights were created using the TWANG macro based on the average treatment effect.^15^ Initial variables considered for inclusion in weights were chosen based on clinical judgment; the final model was chosen based on model parsimony and relative balance of characteristics across the cohort after weighting. Final weights included pretrial characteristics (age, body mass index [kg/m^2^], cocaine use, tobacco use, and UDS for opiates, health insurance, depression [BDI-II], pain [BPI]) in addition to select additional characteristics collected at RECOVER baseline (history of buprenorphine or methadone treatment before trial, and lifetime opioid use [years] before trial).

## RESULTS

### Study Completion

Of 533 RECOVER participants, 529 had at least 1 measurement over the 18-month follow-up, which formed the basis of our cohort (Table 2). A total of 522 participants had outcomes captured at the baseline assessment. Over 70% of participants provided responses at each 6-month assessment (6 months, n = 430; 12 months, n = 417; 18 months, n = 387) with 268 (50%) completing all assessments.

**TABLE 2:** Participant Demographic Characteristics and Baseline Outcomes

	All (N = 529)	0–2 mo BUP-XR (N = 116)	3–5 mo BUP-XR (N = 61)	6–11 mo BUP-XR (N = 86)	12 mo BUP-XR (N = 135)	13–18 mo BUP-XR (N = 131)	*P*
Pretrial Characteristics							
Male	66.2%	61.2%	65.6%	67.4%	68.1%	67.9%	0.778
Age at baseline, mean (SD)	41.8 (11.8)	37.5 (11.2)	38.6 (11.7)	41.4 (10.7)	44.6 (12.0)	44.4 (11.4)	<0.001
Race							
Non-Hispanic White	56.7%	29.7%	29.5%	30.8%	25.6%	27.9%	0.538
Black	28.5%	9.5%	12.3%	11.6%	17.4%	17.9%	0.012
Trial baseline BMI, mean (SD), kg/m^2^	25.7 (4.3)	24.8 (4.0)	25.5 (4.5)	26.1 (4.8)	26.2 (4.3)	25.6 (4.2)	0.109
Pretrial employment	35.2%	19.9%	16.1%	15.5%	18.1%	15.2%	0.788
People who use injectable opioids	45.2%	45.7%	45.9%	40.7%	40.7%	51.9%	0.381
Lifetime opioid use, mean (SD), y	11.7 (9.8)	10.8 (9.1)	9.7 (8.7)	11.4 (9.6)	12.9 (10.2)	12.3 (10.5)	0.156
UDS positive for opiates	67.0%	82.8%	83.6%	61.2%	50.7%	65.9%	<0.001
Additional RECOVER Characteristics (%)							
Education							0.656
<High school	16.3	12.9	16.4	14.0	17.0	19.8	
High school/GED	66.7	67.2	73.8	67.4	67.4	61.8	
College degree or more	17.0	19.8	9.8	18.6	15.6	18.3	
Stable housing	79.0	77.6	72.1	75.6	80.0	84.7	0.271
Age of first nonmedical opioid use ≤16	30.1	32.8	29.5	30.2	29.6	28.2	0.960
Lifetime history of treatment with buprenorphine	36.3	45.7	50.8	30.2	31.1	30.5	0.005
Values for Outcomes at RECOVER Baseline							
Abstinence from opioids							
Past week self-report	64.4%	38.8%	41.0%	68.6%	71.9%	88.7%	<0.001
UDS	63.8%	41.4%	41.8%	70.7%	71.5%	81.3%	<0.001
Self-report + UDS	54.8%	28.4%	31.1%	59.3%	63.0%	79.0%	<0.001
Abstinence from all illicit drugs or misuse of prescriptions	43.7%	25.9%	21.3%	46.5%	47.4%	65.3%	<0.001
Depression (BDI-II), mean (SD)	7.8 (10.5)	11.8 (13.6)	8.8 (8.8)	6.5 (10.7)	6.2 (8.7)	6.3 (8.3)	<0.001
Health-related quality of life (SF-12), mean (SD)							
Physical	49.5 (8.7)	48.5 (9.0)	47.0 (7.5)	49.0 (9.0)	52.9 (6.9)	48.4 (9.4)	<0.001
Mental	43.7 (12.1)	39.1 (12.1)	38.7 (11.9)	43.0 (12.5)	50.0 (10.4)	44.1 (10.6)	<0.001
Employment status	47.7%	43.4%	47.5%	48.8%	49.3%	49.2%	0.889

BDI-II, Brief Depression Index II; BMI, body mass index; BUP-XR, buprenorphine extended release; GED, general educational development; RECOVER, Remission From Chronic Opioid Use: Studying Environmental and Socioeconomic Factors on Recovery; SD, standard deviation; SF-12, 12-Item Short Form Health Survey; UDS, urine drug screen.

A total of 116 participants (21.9%) comprised the 0–2 months, 61 (11.5%) the 3–5 months, 86 (16.3%) the 6–11 months, 135 (25.5%) the 12 months, and 131 (24.8%) the 13–18 months BUP-XR treatment duration groups at the baseline assessment (Table 2). Patients in the 0–2 months and 3–5 months BUP-XR treatment duration groups were least likely to complete all assessments (32.8% and 37.7%, respectively) with higher completion rates across all visits for 6–11 months (60.5%), 12 months (59.3%), and 13–18 months (57.3%) BUP-XR duration groups.

### Participant Characteristics

Overall, 66.2% of participants were male, 56.7% were White, 45.2% had a history of injectable opioid use, and 79.0% lived in stable housing at RECOVER baseline; mean participant age was 41.8 years (Table 2). Key characteristics that differed across BUP-XR duration groups included age, Black race, pretrial positive UDS for opiates, and prior use of buprenorphine (Table 2). After weighting, differences in pretrial positive UDS remained statistically significant with those who eventually received more BUP-XR doses generally being less likely to have tested positive for opioids before their first BUP-XR trial (Supplemental Tables S1 and S2, http://links.lww.com/JAM/A374).

At RECOVER baseline, there were significant differences in outcomes across participants who had received different durations of BUP-XR, and those who had received a longer duration of treatment were more likely to be abstinent and have lower depression scores. Although differences in HRQoL were also statistically significantly different, trends were less clear across BUP-XR duration groups. Employment was 47.7% at RECOVER baseline (Table 2).

### Substance Use Disorder Treatment During RECOVER

Of the 411 participants who provided information on substance use disorder (SUD) treatment at any semiannual assessment during the 18-month follow-up period, 32.8% reported receiving pharmacotherapy for OUD over follow-up (46.4% of 0–2 months, 42.2% of 3–5 months, 21.4% of 6–11 months, 30.1% of 12 months, and 30.6% of 13–18 months BUP-XR treatment duration groups). Buprenorphine was the most common medication used (27.4%), followed by methadone (6.1%) and naltrexone (1.2%); participants could have used more than one of these during the follow-up period. Other treatments included counseling (16.2%) and 12-step programs (18.7%). The proportion of participants receiving treatment increased over time during the RECOVER follow-up period (Table 3). For those who did not report receiving any treatment for SUD at any assessment for the entire 18-month observational period (n = 178), 94.4% reported that one reason for this was that they did not feel they needed treatment. Other reasons included not being able to afford treatment (15.9%), not wanting anyone to know (8.5%), and insurance concerns (8.5%).

**TABLE 3:** Pharmacotherapy and Changes in Outcomes Over Time Within the RECOVER Cohort

	All Participants (%)			Excluding Patients with any MOUD		
	6 mo	12 mo	18 mo	6 mo	12 mo	18 mo
Any pharmacotherapy for opioid use disorder	13.4	17.8	24.3	—	—	—
Buprenorphine	11.0	14.9	19.4	—	—	—
Methadone	1.9	3.9	4.9	—	—	—
Naltrexone	0.5	0.3	0.6	—	—	—
Abstinence						—
Abstinence from opioids (past week self-report)						
Improved, abstinent	14.4	14.6	16.8	18.7	11.9	15.5
Stable, abstinent	51.8	52.3	52.1	52.2	51.1	47.4
Stable, nonabstinent	20.5	20.9	17.4	19.2	24.5	21.6
Declined, nonabstinent	13.4	12.2	13.7	9.8	12.4	15.5
Abstinence from all illicit substances or misuse of prescription medications*						
Improved, abstinent	14.1	15.3	21.4	19.9	15.4	20.2
Stable, abstinent	31.3	31.9	31.7	28.4	28.2	27.4
Stable, nonabstinent	42.4	41.4	34.3	41.8	44.0	38.7
Declined, nonabstinent	12.2	11.4	12.7	9.8	12.4	13.8
Health-related quality of life (SF-12)						
PCS						
Improved	22.7	28.0	23.9	25.3	28.6	24.5
Stable	45.4	44.2	37.8	45.2	44.0	39.9
Declined	31.8	27.8	38.3	29.4	27.4	35.6
MCS						
Improved	24.0	20.6	19.5	27.6	15.0	18.0
Stable	50.6	45.1	43.0	49.3	50.8	44.6
Declined	25.4	34.3	37.6	23.0	34.2	37.4
Depression (BDI-II)						
Improved	21.6	20.9	20.9	22.3	19.5	17.6
Stable	53.4	55.2	55.0	55.4	55.1	58.5
Declined	25.1	23.9	24.1	22.3	25.4	23.9
Employment						
Improved, employed	8.8	11.4	11.3	14.6	15.9	11.2
Stable, employed	36.3	36.3	35.5	32.7	34.6	37.4
Stable, unemployed	43.9	41.5	41.7	40.3	36.8	40.4
Declined, unemployed	11.0	10.7	11.6	12.4	12.6	11.0

*Excluding cannabis.

BDI-II, Brief Depression Index II; MCS, Mental Component Summary Score; MOUDs, medications for opioid use disorder; PCS, Physical Component Summary; RECOVER, Remission From Chronic Opioid Use: Studying Environmental and Socioeconomic Factors on Recovery; SF-12, 12-Item Short Form Health Survey.

### Abstinence From Opioids and Illicit Substances

Within the full cohort, 46.9% of the 480 participants for whom abstinence over time could be calculated had sustained abstinence for the entire 18-month observation period. Sustained abstinence over the entire 18-month follow-up period was statistically significantly higher (*P* < 0.001) for the 6–11 months (47.5%), 12 months (52.1%), and 13–18 months (62.5%) BUP-XR treatment duration groups compared with the 0–2 months (36.6%) BUP-XR treatment duration group, but lower for the 3–5 months BUP-XR duration group (24.0%) after weighting, indicating higher abstinence in those who received more injections.

**Figure 2:**
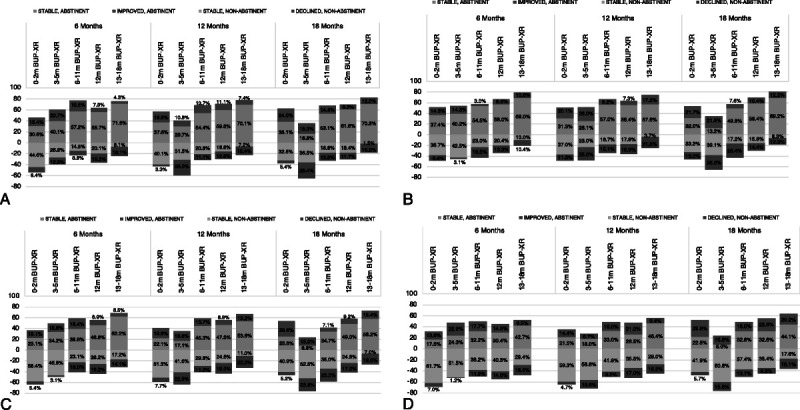
Abstinence-related outcomes over 18-month follow-up period, by BUP-XR treatment duration group. A, Self-reported abstinence from opioids rates. B, UDS-based abstinence from opioids rates. C, Combined self-reported and UDS-based abstinence from opioids rates. D, Self-reported abstinence from all illicit substances rates. Abbreviations: BUP-XR, buprenorphine extended release; m, months; UDS, urine drug screen. Results represent weighted proportion of participants, with changes measured change from RECOVER baseline. Definitions of improved, stable, and declined were based on movement from abstinent to nonabstinent as decline, or movement from nonabstinent to abstinent as improvement. Inverse probability weights included key pretrial characteristics as described within text. Proportions for those who declined were multiplied by −1 for graphing purposes to aid in the easy interpretation of figures.

Out of the entire cohort, 66.2% of participants were abstinent at 6 months (14.4% becoming abstinent after baseline; 51.8% stable abstinent), 66.9% at 12 months, and 68.9% at 18 months (Table 3) based on past week self-report. Similar results were found when excluding patients with any MOUD; 70.9% of participants were abstinent at 6 months, 63% at 12 months, and 62.9% at 18 months (Table 3). After weighting and over the entire cohort, there were statistically significant differences in the proportion of respondents who were abstinent at the 6-, 12-, and 18-month visits across BUP-XR duration groups (Fig. 2A; Supplemental Table S1, http://links.lww.com/JAM/A374; *P* < 0.001). The greatest amount of improvement in abstinence occurred within the 0–2 months and 3–5 months BUP-XR treatment duration groups, but this may have also had to do with lower proportions of participants who were abstinent at baseline. Similar trends were observed when evaluating abstinence based on UDS (Fig. 2B; Supplemental Table S1, http://links.lww.com/JAM/A374; *P* < 0.05 at 6- and 18-month visits) and combining UDS and self-reported information (Fig. 2C; Supplemental Table S1, http://links.lww.com/JAM/A374).

Concerning other substances, over the total study period, 64.1% of participants reported using nonmedical cannabis, 43.1% reported using cocaine, and 83.2% reported using a nonopioid illicit drug or misusing nonopioid prescription medication. In the full cohort, 45.4% of respondents were abstinent from illicit substances (excluding cannabis) at 6 months, 47.2% were abstinent at 12 months, and 53.0% were abstinent at 18 months (Table 3). Excluding patients with any MOUD, 48.3% of respondents were abstinent from illicit substances (excluding cannabis) at 6 months, 43.6% at 12 months, and 47.6% at 18 months (Table 3). Over the entire cohort, there were statistically significant differences in the proportion of patients who were abstinent across BUP-XR treatment duration groups at 12 months (*P* = 0.025) and 18 months (*P* < 0.001), and statistically significantly different patterns of change at 6, 12, and 18 months (*P* < 0.05; Fig. 2D; Supplemental Table S1, http://links.lww.com/JAM/A374).

### Health-related Quality of Life and Psychological Distress

Out of the entire cohort, at 6 months, 68.1% of participants had improved or stable SF-12 PCS, with 72.2% improved/stable at 12 months and 61.7% improved/stable at 18 months (Table 3). Similarly, excluding patients with any MOUD, 70.5% of participants had improved or stable PCS at 6 months, 72.6% at 12 months, and 64.4% at 18 months (Table 3).

SF-12 MCS showed similar results over the entire cohort, with 74.6% improved/stable at 6 months, 65.7% at 12 months, and 62.5% at 18 months (Table 3). Excluding patients with any MOUD, 76.9% of participants at 6 months, 65.8% at 12 months, and 62.6% at 18 months had improved or stable MCS (Table 3).

Over the entire cohort, there were statistically significant differences in change patterns for PCS at 12 months, with the 3–5 months BUP-XR treatment duration group least likely to decline and 12 months BUP-XR treatment duration group most likely to decline (Supplemental Fig. S1E and Table S1, http://links.lww.com/JAM/A374). For MCS, there were only statistically significant differences across treatment arms at 6 months, with the 3–5 months BUP-XR treatment duration group more likely to improve than other groups (Supplemental Fig. S1F and Table S1, http://links.lww.com/JAM/A374).

In the entire cohort, 75% had stable or improved BDI-II scores at 6 months, 76.1% at 12 months, and 75.9% at 18 months (Table 3). Similarly, excluding patients with any MOUD, 77.7% showed stable or improved BDI-II scores at 6 months, 74.6% at 12 months, and 76.1% at 18 months (Table 3). After weighting, over the entire cohort, there were statistically significant differences in patterns of change at 12 months (*P* = 0.025), with participants who were in the 13–18 months BUP-XR treatment duration group more likely to be improved or stable compared with shorter treatment duration groups (Supplemental Fig. S1G and Table S1, http://links.lww.com/JAM/A374).

### Employment

Most patients either maintained their baseline employment or moved from unemployed to employed during the RECOVER follow-up period (in the entire cohort: 45.1% employed at 6 months, 47.7% at 12 months, 46.8% at 18 months; excluding patients with any MOUD: 47.3% employed at 6 months, 50.5% at 12 months, 48.6% at 18 months; Table 3). For all BUP-XR treatment duration groups, although employment remained relatively stable over time, the largest difference in change patterns across BUP-XR groups was at 6 months (*P* = 0.05) after weighting the cohort. At the 6-month time point, participants in the 6–11 months BUP-XR treatment duration group were the most likely to become unemployed, and those in the 13–18 months BUP-XR treatment duration group were the least likely (Supplemental Fig. S1H and Supplemental Table S1, http://links.lww.com/JAM/A374).

## DISCUSSION

Up to 18 months after the last BUP-XR injection, RECOVER study participants seemed to largely retain or improve in their outcomes over time, and those receiving a longer duration of BUP-XR had better outcomes. Almost half of participants reported sustained abstinence for 18 months after discontinuation of BUP-XR within a clinical trial setting.

Our results are consistent with a meta-analysis^16^ showing that benefits from sustained treatment can extend beyond the period of active treatment itself. Although the majority of the studies summarized within the meta-analysis were in people who use heroin, the RECOVER cohort was composed of participants who have a varied opioid use background,^7^ with 65% using both heroin and prescription opioids in their history.^17^

Longer BUP-XR treatment duration was associated with higher rates of abstinence from opioids and other illicit drug use, lower depression, and better HRQoL outcomes at RECOVER baseline, with equivalent or better rates of stability or improvement across BUP-XR treatment duration groups. Our results may be indicative of the BUP-XR safety and efficacy profile; potential advantages include consistent protection with sustained plasma concentrations (2–3 ng/mL or above) during monthly treatment and potentially longer duration effects, as buprenorphine concentrations slowly decrease after discontinuation of treatment.^18–20^

This finding is consistent with evidence on treatment duration with buprenorphine, indicating significant improvements in patient outcomes for 15 months compared with 6–9 months of treatment.^21^ The Vivitrol Cost and Treatment Outcomes Registry analysis, measuring the impact of extended-release naltrexone on patient-relevant outcomes, also suggests that longer treatment may be associated with better outcomes.^17^ RECOVER also demonstrates that positive outcomes may extend beyond the initial treatment period. Participants’ health, economic, and social benefits while on active BUP-XR treatment during clinical trials were maintained during the RECOVER follow-up period.^6,7^ The relationship between BUP-XR treatment duration before RECOVER and other patient-reported outcomes was less apparent than those with abstinence outcomes. This is not surprising because the casual link between pharmacotherapy and abstinence is more proximal than clinical, psychosocial, and economic outcomes.

This study has several limitations. Participants were not randomized to BUP-XR treatment duration groups, and although weighted analyses were applied, comparisons between BUP-XR treatment duration groups were potentially biased. Findings may be subject to floor and ceiling effects, as participants receiving the fewest BUP-XR doses had better outcomes during the follow-up period, even though their overall performance may not have been as good as those receiving more doses. This illustrates that there may be different paths to recovery. In addition, decreased completion rates for shorter-duration groups may add potential bias.

Although we weighted the treatment duration groups to reflect similar pretrial characteristics, there may have been residual differences. Length of time between last BUP-XR dose and enrollment in the RECOVER study and differences between study completers and participants who discontinued prematurely may have affected comparisons across treatment duration groups. In addition, almost one third of participants reported pharmacotherapy use for OUD as well during the RECOVER follow-up period. However, when we removed any patients receiving MOUDs during the follow-up period, we found similar results. Given these limitations, it is not possible to assign a causative relationship between BUP-XR treatment duration and study outcomes.

A strength of this study is the use of both self-report and UDS to assess abstinence. There was a high correlation between self-reported and UDS results within this cohort,^7^ and sensitivity analysis yielded similar findings regardless of outcome measure. No OUD treatment was given to participants as part of the RECOVER study, but knowledge of being within the RECOVER study and being asked to complete a survey quarterly may have modified behavior (ie, Hawthorne effect^22^).

To advance understanding of personalized treatment duration, it is important to understand why those with shorter duration of treatment can achieve abstinence. The ongoing RECOVER cohort follow-up (NCT04577144) would provide a greater perspective of long-term outcomes in treatment-seeking participants with OUD. The generalizability of these results to individuals who initiate treatment with BUP-XR outside of a clinical trial treatment setting warrants further study.

## CONCLUSIONS

Over an 18-month time frame after RECOVER BUP-XR trial participation, a large proportion of participants achieved and retained favorable abstinence, psychosocial, and clinical outcomes. Additional evaluation of these endpoints over time is needed.

## Supplementary Material
