# Supplementary material for: Continued Posttrial Benefits of Buprenorphine Extended Release: RECOVER Study Findings
Source: J Addict Med. 2022 Sep 16;17(2):182–9. doi: 10.1097/ADM.0000000000001070 (PMC10022675; doi:10.1097/ADM.0000000000001070)
Supplement: Supplementary file 2 [file jam-17-182-s002.docx]

# Supplemental Digital Content

Supplemental Table 1. Participant Demographic Characteristics by BUP-XR Treatment Duration, Before and After Weighting

|  | | **Unweighted and Weighted Results** | | | | | | | | | | | | | |
| --- | --- | --- | --- | --- | --- | --- | --- | --- | --- | --- | --- | --- | --- | --- | --- |
|  | | **Weighted result** | | | | | | | **Unweighted result** | | | | | | |
| **Variable** | **Statistic or Category** | **All** | **0-2m BUP-XR** | **3-5m BUP-XR** | **6-11m BUP-XR** | **12m BUP-XR** | **13-18m BUP-XR** | ***P* value** | **All** | **0-2m BUP-XR** | **3-5m BUP-XR** | **6-11m BUP-XR** | **12m BUP-XR** | **13-18m BUP-XR** | ***P* value** |
|  |  | **(N = 339)** | **(N = 92)** | **(N = 40)** | **(N = 75)** | **(N = 92)** | **(N = 67)** |  | **(N = 529)** | **(N = 116)** | **(N = 61)** | **(N = 86)** | **(N = 135)** | **(N = 131)** |  |
| **Pre-trial Characteristics** | | | | | | | | | | | | | | | |
| **Biological Sex, %** | Male | 65.0 | 59.8 | 65.1 | 67.7 | 62.5 | 70.2 | 0.766^C^ | 66.2 | 61.2 | 65.6 | 67.4 | 68.1 | 67.9 | 0.778^C^ |
|  | Female | 35.0 | 40. | 34.9 | 32.3 | 37.5 | 29.8 |  | 33.8 | 38.8 | 34.4 | 32.6 | 31.9 | 32.1 |  |
| **Age at Baseline** | Mean (SD) | 41.4 (14.9) | 39.3 (14.0) | 41.9 (17.3) | 41.5 (11.7) | 41.8 (13.0) | 42.2 (14.5) | 0.541^R^ | 41.8 (11.8) | 37.5 (11.2) | 38.6 (11.7) | 41.4 (10.7) | 44.6 (12.0) | 44.4 (11.4) | <0.001^R^ |
| **Race, %** | Non-Hispanic White | 58.1 | 29.1 | 31.3 | 30.7 | 26.5 | 27.9 | 0.756^C^ | 56.7 | 29.7 | 29.5 | 30.8 | 25.6 | 27.9 | 0.538^C^ |
|  | Black | 25.2 | 9.6 | 12.8 | 11.7 | 13.9 | 14.7 | 0.683^C^ | 28.5 | 9.5 | 12.3 | 11.6 | 17.4 | 17.9 | 0.012^C^ |
|  | Other | 16.7 | 61.3 | 55.9 | 57.6 | 59.6 | 57.4 |  |  | 60.8 | 58.2 | 57.6 | 57.0 | 54.2 |  |
| **Trial Baseline BMI (kg/m2)** | Mean (SD) | 25.5 (4.6) | 25.1 (4.3) | 25.3 (4.1) | 25.7 (4.4) | 25.8 (5.0) | 25.7 (4.1) | 0.708^R^ | 25.7 (4.3) | 24.8 (4.0) | 25.5 (4.5) | 26.1 (4.8) | 26.2 (4.3) | 25.6 (4.2) | 0.109^R^ |
| **Pre-trial Employment, %** | No | 64.3 | 31.6 | 32.4 | 33.3 | 30.9 | 33.6 | 0.978^C^ | 64.8 | 30.1 | 33.9 | 34.5 | 31.9 | 34.8 | 0.788^C^ |
|  | Yes | 35.7 | 18.4 | 17.6 | 16.7 | 19.1 | 16.4 |  | 35.2 | 19.9 | 16.1 | 15.5 | 18.1 | 15.2 |  |
| **Pre-trial tobacco use, %** | Current | 85.0 | 82.3 | 75.0 | 87.3 | 88.1 | 90.7 | 0.203^C^ | 84.7 | 80.2 | 80.3 | 87.2 | 84.4 | 89.3 | 0.123^C^ |
|  | Former | 5.9 | 8.7 | 9.5 | 3.1 | 6.3 | 2.4 |  | 6.8 | 12.9 | 8.2 | 3.5 | 6.7 | 3.1 |  |
|  | Never | 9.2 | 9.0 | 15.5 | 9.6 | 5.6 | 6.9 |  | 8.5 | 6.9 | 11.5 | 9.3 | 8.9 | 7.6 |  |
| **Alcohol use at screen, %** | Current | 51.9 | 55.1 | 47.7 | 54.4 | 54.5 | 46.3 | 0.683^C^ | 51.2 | 54.3 | 42.6 | 54.7 | 50.4 | 51.1 | 0.611^C^ |
|  | Former | 24.7 | 22.8 | 22.5 | 20.4 | 25.4 | 33.5 |  | 25.3 | 25.0 | 26.2 | 18.6 | 28.9 | 26.0 |  |
|  | Never | 23.4 | 22.1 | 29.8 | 25.1 | 20.1 | 20.2 |  | 23.4 | 20.7 | 31.1 | 26.7 | 20.7 | 22.9 |  |
| **Pre-trial cocaine use, %** | No | 65.9 | 74.0 | 62.5 | 64.9 | 64.4 | 65.1 | 0.707^C^ | 64.8 | 72.4 | 68.9 | 64.0 | 64.4 | 57.3 | 0.153^C^ |
|  | Yes | 34.1 | 26.0 | 37.5 | 35.1 | 35.6 | 34.9 |  | 35.2 | 27.6 | 31.1 | 36.0 | 35.6 | 42.7 |  |
| **People Who Use Injectable Opioids (All Phase 3), %** | People Who Use Injectable Opioids | 44.7 | 40.3% | 44.6% | 38.9% | 46.0% | 54.7% | 0.398^C^ | 45.2% | 45.7% | 45.9% | 40.7% | 40.7% | 51.9% | 0.381^C^ |
|  | People Who Use Noninjectable Opioids | 55.3 | 59.7% | 55.4% | 61.1% | 54.0% | 45.3% |  | 54.8% | 54.3% | 54.1% | 59.3% | 59.3% | 48.1% |  |
| **Lifetime Opioid Use (Years) (All Phase 3)** | Mean (SD) | 11.1 (10.8) | 10.5 (9.1) | 11.7 (10.7) | 10.7 (9.1) | 11.5 (10.3) | 10.9 (12.6) | 0.900^R^ | 11.7 (9.8) | 10.8 (9.1) | 9.7 (8.7) | 11.4 (9.6) | 12.9 (10.2) | 12.3 (10.5) | 0.156^R^ |
| **Pre-trial urine drug screen for opiates, %** | Negative | 28.9 | 22.2 | 14.2 | 33.1 | 37.1 | 34.5 | 0.008^C^ | 33.0 | 17.2 | 16.4 | 38.8 | 49.3 | 34.1 | <0.001^C^ |
|  | Positive | 71.1 | 77.8 | 85.8 | 66.9 | 62.9 | 65.5 |  | 67.0 | 82.8 | 83.6 | 61.2 | 50.7 | 65.9 |  |
| **Additional RECOVER Characteristics** | | | | | | | | | | | | | | | |
| **Education, %** | <HS | 16.3 | 14.5 | 17.6 | 14.6 | 16.5 | 18.7 | 0.873^C^ | 16.3 | 12.9 | 16.4 | 14.0 | 17.0 | 19.8 | 0.656^C^ |
|  | HS/GED | 67.4 | 65.0 | 72.7 | 66.8 | 67.7 | 64.3 |  | 66.7 | 67.2 | 73.8 | 67.4 | 67.4 | 61.8 |  |
|  | College degree or more | 16.3 | 20.5 | 9.7 | 18.6 | 15.8 | 17.0 |  | 17.0 | 19.8 | 9.8 | 18.6 | 15.6 | 18.3 |  |
| **Stable housing, %** | No | 21.4 | 20.8 | 24.8 | 20.9 | 25.1 | 13.3 | 0.422^C^ | 21.0 | 22.4 | 27.9 | 24.4 | 20.0 | 15.3 | 0.271^C^ |
|  | Yes | 78.6 | 79.2 | 75.2 | 79.1 | 74.9 | 86.7 |  | 79.0 | 77.6 | 72.1 | 75.6 | 80.0 | 84.7 |  |
| **Age of first non-medical opioid use ≤16, %** | No | 69.8 | 71.7 | 74.6 | 71.1 | 60.6 | 74.0 | 0.244^C^ | 69.9 | 67.2 | 70.5 | 69.8 | 70.4 | 71.8 | 0.960^C^ |
|  | Yes | 30.2 | 28.3 | 25.4 | 28.9 | 39.4 | 26.0 |  | 30.1 | 32.8 | 29.5 | 30.2 | 29.6 | 28.2 |  |
| **Lifetime History of treatment with buprenorphine, %** | No | 62.0 | 54.1 | 54.7 | 68.8 | 67.0 | 61.8 | 0.283^C^ | 63.7 | 54.3 | 49.2 | 69.8 | 68.9 | 69.5 | 0.005^C^ |
|  | Yes | 38.0 | 45.9 | 45.3 | 31.2 | 33.0 | 38.2 |  | 36.3 | 45.7 | 50.8 | 30.2 | 31.1 | 30.5 |  |
| **Values for Outcomes at RECOVER Baseline** | | | | | | | | | | | | | | | |
| **Abstinence from opioids (Past week self-report), %** | Abstinent | 63.3 | 42.0 | 46.5 | 67.9 | 71.2 | 88.5 | <0.001^C^ | 64.4 | 38.8 | 41.0 | 68.6 | 71.9 | 88.7 | <0.001^C^ |
|  | Non-abstinent | 36.7 | 58.0 | 53.5 | 32.1 | 28.8 | 11.5 |  | 35.6 | 61.2 | 59.0 | 31.4 | 28.1 | 11.3 |  |
| **Abstinence from opioids (UDS), %** | Abstinent | 62.3 | 45.2 | 38.5 | 74.3 | 70.4 | 80.8 | <0.001^C^ | 63.8 | 41.4 | 41.8 | 70.7 | 71.5 | 81.3 | <0.001^C^ |
|  | Non-abstinent | 37.7 | 54.8 | 61.5 | 25.7 | 29.6 | 19.2 |  | 36.2 | 58.6 | 58.2 | 29.3 | 28.5 | 18.8 |  |
| **Abstinence from opioids (Self Report + UDS), %** | Abstinent | 53.8 | 31.5 | 33.1 | 59.8 | 64.2 | 78.1 | <0.001^C^ | 54.8 | 28.4 | 31.1 | 59.3 | 63.0 | 79.0 | <0.001^C^ |
|  | Non-abstinent | 46.2 | 68.5 | 66.9 | 40.2 | 35.8 | 21.9 |  | 45.2 | 71.6 | 68.9 | 40.7 | 37.0 | 21.0 |  |
| **Abstinence from all illicit drugs or misuse of prescriptions, %** | Abstinent | 41.6 | 27.6 | 25.2 | 46.5 | 45.8 | 63.6 | <0.001^C^ | 43.7 | 25.9 | 21.3 | 46.5 | 47.4 | 65.3 | <0.001^C^ |
|  | Non-abstinent | 58.4 | 72.4 | 74.8 | 53.5 | 54.2 | 36.4 |  | 56.3 | 74.1 | 78.7 | 53.5 | 52.6 | 34.7 |  |
| **Depression (BDI-II)** | Mean (SD) | 8.0 (12.9) | 11.9 (14.3) | 8.6 (9.7) | 6.5 (12.6) | 7.2 (13.7) | 6.6 (9.2) | 0.013^R^ | 7.8 (10.5) | 11.8 (13.6) | 8.8 (8.8) | 6.5 (10.7) | 6.2 (8.7) | 6.3 (8.3) | <0.001^R^ |
| **Physical HRQoL (SF-12 PCS)** | Mean (SD) | 49.5 (10.0) | 48.3 (10.3) | 48.7 (8.1) | 49.0 (9.4) | 51.4 (11.1) | 49.1 (9.7) | 0.183^R^ | 49.5 (8.7) | 48.5 (9.0) | 47.0 (7.5) | 49.0 (9.0) | 52.9 (6.9) | 48.4 (9.4) | <0.001^R^ |
| **Mental HRQoL (SF-12 MCS)** | Mean (SD) | 43.0 (14.8) | 39.3 (14.2) | 40.6 (14.2) | 42.9 (14.5) | 46.5 (15.2) | 44.7 (11.4) | <0.001^R^ | 43.7 (12.1) | 39.1 (12.1) | 38.7 (11.9) | 43.0 (12.5) | 50.0 (10.4) | 44.1 (10.6) | <0.001^R^ |
| **Probable Serious Mental Illness (K6, SDS), %** | No probable serious mental illness | 89.6 | 84.6 | 90.0 | 89.7 | 91.2 | 92.2 | 0.696^C^ | 90.8 | 86.1 | 88.5 | 90.6 | 94.1 | 92.7 | 0.221^C^ |
|  | Probable serious Mental Illness | 10.4 | 15.4 | 10.0 | 10.3 | 8.8 | 7.8 |  | 9.2 | 13.9 | 11.5 | 9.4 | 5.9 | 7.3 |  |
| **Employment Status, %** | Employed | 48.3 | 40.8 | 45.9 | 51.7 | 50.4 | 50.7 | 0.686^C^ | 47.7 | 43.4 | 47.5 | 48.8 | 49.3 | 49.2 | 0.889^C^ |
|  | Unemployed | 51.7 | 59.2 | 54.1 | 48.3 | 49.6 | 49.3 |  | 52.3 | 56.6 | 52.5 | 51.2 | 50.7 | 50.8 |  |

^a^ This represents the re-weighted pseudo-n after weighting.

^b^ Variable was included in inverse probability weight model

Abbreviations: BDI-II, Brief Depression Index-II; BMI, body mass index; BUP-XR, buprenorphine extended release; C, chi-squared based p-value; K6, Kessler’s psychological distress scale; HRQoL, Health-related quality of life; m, months; R, Regression-based p-value; SF-12 MCS, 12-item short form mental component summary; SF-12 PCS, 12 item short form physical component summary; SDS, Sheehan disability scale; UDS, urine drug screen.

Supplemental Figure 1. Weighted and Unweighted Changes in Outcomes by BUP-XR Treatment Duration Group

1. Abstinence from opioids based on past week self-report

1. Change in abstinence from opioids based on urine drug screen
2. Change in abstinence from opioids based on both past week self-report and urine drug screen
3. Abstinence from all illicit drugs or misuse of prescription medications
4. Physical Health-related Quality of Life (SF-12 Physical Component Summary)
5. Mental Health-related Quality of Life (SF-12 Mental Component Summary)
6. Depression (BDI-II)
7. Employment

Results represent weighted proportion of participants measuring change from RECOVER baseline. Definitions of improved, stable, and declined were based on movement from a negative to a positive state (e.g., abstinent to non-abstinent as decline, or movement from non-abstinent to abstinent as improvement) or based on improvements or declines greater than minimally clinically important differences as defined in Table 1. Inverse probability weights included key pre-trial characteristics as described within text. Proportions for those who declined were multiplied by -1 for graphing purposes to aid in the easy interpretation of figures.

Abbreviations: BDI-II, Brief Depression Index; BUP-XR, buprenorphine extended release; m, months; SF-12, 12-Item Short Form Health Survey.

Supplemental Table 2. Participant Demographic Characteristics by BUP-XR Treatment Duration, Before and After Weighting, Removing Subjects Who Received Any MOUD During RECOVER

|  | | **Unweighted and Weighted Results** | | | | | | | | | | | |
| --- | --- | --- | --- | --- | --- | --- | --- | --- | --- | --- | --- | --- | --- |
|  | | **Weighted Result** | | | | | | **Unweighted Result** | | | | | |
| **Variable** | **Statistic or Category** | **All** | **0-2m BUP-XR** | **3-5m BUP-XR** | **6-11m BUP-XR** | **12-18m BUP-XR** | ***P* Value** | **All** | **0-2m BUP-XR** | **3-5m BUP-XR** | **6-11m BUP-XR** | **12-18m BUP-XR** | ***P* Value** |
|  |  | **(N = 196)** | **(N = 65)** | **(N = 32)** | **(N = 63)** | **(N = 63)** |  | **(N = 297)** | **(N = 79)** | **(N = 49)** | **(N = 73)** | **(N = 96)** |  |
| **Biological sex, N (%)** | Male | 63.8 | 63.4 | 62.4 | 68.9 | 59.1 | 0.754^C^ | 65.7 | 63.3 | 63.3 | 68.5 | 66.7 | 0.891^C^ |
|  | Female | 36.2 | 36.6 | 37.6 | 31.1 | 40.9 |  | 34.3 | 36.7 | 36.7 | 31.5 | 33.3 |  |
| **Age at baseline** | Mean (SD) | 41.0 (15.6) | 38.1 (13.6) | 42.5 (17.6) | 41.1 (12.3) | 41.4 (12.8) | 0.286^R^ | 40.5 (11.9) | 36.5 (10.7) | 38.5 (12.1) | 40.9 (11.1) | 44.5 (12.2) | <0.001^R^ |
| **Non-Hispanic White, N (%)** | No | 44.6 | 27.5 | 19.1 | 20.4 | 24.1 | 0.364^C^ | 46.1 | 25.3 | 20.4 | 19.9 | 25.0 | 0.394^C^ |
|  | Yes | 55.4 | 22.5 | 30.9 | 29.6 | 25.9 |  | 53.9 | 24.7 | 29.6 | 30.1 | 25.0 |  |
| **Black, N (%)** | No | 72.7 | 36.4 | 36.1 | 37.6 | 35.0 | 0.943^C^ | 70.7 | 37.3 | 36.7 | 37.7 | 31.3 | 0.198^C^ |
|  | Yes | 27.3 | 13.6 | 13.9 | 12.4 | 15.0 |  | 29.3 | 12.7 | 13.3 | 12.3 | 18.8 |  |
| **Baseline BMI (kg/m^2^)** | Mean (SD) | 25.6 (4.7) | 25.0 (4.3) | 25.5 (4.2) | 25.6 (4.4) | 25.9 (5.4) | 0.685^R^ | 25.7 (4.4) | 24.9 (3.9) | 25.7 (4.7) | 25.9 (4.8) | 26.2 (4.4) | 0.235^R^ |
| **Pre-trial employment, N (%)** | No | 65.8 | 32.9 | 35.8 | 31.5 | 33.3 | 0.917^C^ | 65.7 | 31.6 | 35.0 | 32.7 | 33.3 | 0.948^C^ |
|  | Yes | 34.2 | 17.1 | 14.2 | 18.5 | 16.7 |  | 34.3 | 18.4 | 15.0 | 17.3 | 16.7 |  |
| **Pre-trial tobacco use, N (%)** | Current | 83.3 | 84.2 | 70.3 | 89.8 | 87.6 | 0.107^C^ | 83.8 | 83.5 | 75.5 | 90.4 | 83.3 | 0.443^C^ |
|  | Former | 6.4 | 6.1 | 11.2 | 2.4 | 6.6 |  | 7.1 | 8.9 | 10.2 | 2.7 | 7.3 |  |
|  | Never | 10.3 | 9.7 | 18.4 | 7.9 | 5.8 |  | 9.1 | 7.6 | 14.3 | 6.8 | 9.4 |  |
| **Alcohol use at screen, N (%)** | Current | 51.7 | 50.4 | 47.5 | 56.0 | 51.7 | 0.785^C^ | 49.5 | 48.1 | 42.9 | 56.2 | 49.0 | 0.372^C^ |
|  | Former | 23.1 | 28.0 | 20.4 | 19.6 | 26.7 |  | 26.3 | 30.4 | 24.5 | 17.8 | 30.2 |  |
|  | Never | 25.1 | 21.6 | 32.0 | 24.3 | 21.7 |  | 24.2 | 21.5 | 32.7 | 26.0 | 20.8 |  |
| **Pre-trial cocaine use, N (%)** | No | 63.5 | 66.2 | 61.7 | 64.8 | 61.8 | 0.963^C^ | 65.0 | 67.1 | 69.4 | 63.0 | 62.5 | 0.812^C^ |
|  | Yes | 36.5 | 33.8 | 38.3 | 35.2 | 38.2 |  | 35.0 | 32.9 | 30.6 | 37.0 | 37.5 |  |
| People who use **injectable opioids (all phase 3), N (%)** | People who use injectable opioids | 42.7 | 41.4 | 43.5 | 39.7 | 46.4 | 0.901^C^ | 42.4 | 45.6 | 42.9 | 42.5 | 39.6 | 0.887^C^ |
|  | People who use noninjectable opioids | 57.3 | 58.6 | 56.5 | 60.3 | 53.6 |  | 57.6 | 54.4 | 57.1 | 57.5 | 60.4 |  |
| **Lifetime opioid use, years (all phase 3)** | Mean (SD) | 11.0 (10.5) | 10.2 (8.3) | 12.6 (10.9) | 10.0 (8.7) | 11.4 (10.7) | 0.475^R^ | 11.3 (9.3) | 10.6 (8.4) | 10.5 (9.1) | 10.7 (9.0) | 12.7 (10.3) | 0.415^R^ |
| **Pre-trial urine drug screen for opiates,  N (%)** | Negative | 27.6 | 15.8 | 14.6 | 34.3 | 40.9 | 0.003^C^ | 33.6 | 12.7 | 16.3 | 40.3 | 54.7 | <0.001^C^ |
|  | Positive | 72.4 | 84.2 | 85.4 | 65.7 | 59.1 |  | 66.4 | 87.3 | 83.7 | 59.7 | 45.3 |  |
| **Education, N (%)** | <HS | 17.7 | 19.9 | 18.6 | 16.2 | 17.1 | 0.849^C^ | 17.2 | 16.5 | 18.4 | 15.1 | 18.8 | 0.870^C^ |
|  | HS/GED | 67.5 | 62.4 | 71.9 | 65.0 | 69.5 |  | 68.0 | 65.8 | 71.4 | 67.1 | 68.8 |  |
|  | College degree or more | 14.8 | 17.7 | 9.5 | 18.7 | 13.4 |  | 14.8 | 17.7 | 10.2 | 17.8 | 12.5 |  |
| **Stable housing, N (%)** | No | 22.0 | 19.5 | 27.1 | 17.4 | 24.2 | 0.567^C^ | 22.9 | 21.5 | 30.6 | 21.9 | 20.8 | 0.571^C^ |
|  | Yes | 78.0 | 80.5 | 72.9 | 82.6 | 75.8 |  | 77.1 | 78.5 | 69.4 | 78.1 | 79.2 |  |
| **Age of first non-medical opioid use ≤16, N (%)** | No | 71.8 | 74.4 | 78.5 | 71.5 | 63.6 | 0.321^C^ | 70.4 | 68.4 | 73.5 | 68.5 | 71.9 | 0.895^C^ |
|  | Yes | 28.2 | 25.6 | 21.5 | 28.5 | 36.4 |  | 29.6 | 31.6 | 26.5 | 31.5 | 28.1 |  |
| **LF TX buprenorphine, N (%)** | No | 63.4 | 56.0 | 54.9 | 68.0 | 71.2 | 0.227^C^ | 63.0 | 53.2 | 49.0 | 68.5 | 74.0 | 0.004^C^ |
|  | Yes | 36.6 | 44.0 | 45.1 | 32.0 | 28.8 |  | 37.0 | 46.8 | 51.0 | 31.5 | 26.0 |  |
| **Abstinence from opioids (past week self-report), N (%)** | Abstinent | 59.5 | 40.2 | 44.7 | 69.6 | 75.3 | <0.001^C^ | 57.9 | 39.2 | 38.8 | 69.9 | 74.0 | <0.001^C^ |
|  | Non-abstinent | 40.5 | 59.8 | 55.3 | 30.4 | 24.7 |  | 42.1 | 60.8 | 61.2 | 30.1 | 26.0 |  |
| **Abstinence from opioids (UDS), N (%)** | Abstinent | 59.1 | 43.6 | 36.4 | 75.7 | 73.2 | <0.001^C^ | 60.0 | 44.1 | 40.0 | 71.2 | 74.4 | <0.001^C^ |
|  | Non-abstinent | 40.9 | 56.4 | 63.6 | 24.3 | 26.8 |  | 40.0 | 55.9 | 60.0 | 28.8 | 25.6 |  |
| **Abstinence from opioids (self-report + UDS), N (%)** | Abstinent | 50.0 | 29.6 | 32.2 | 61.0 | 68.5 | <0.001^C^ | 48.8 | 29.1 | 30.6 | 60.3 | 65.6 | <0.001^C^ |
|  | Non-abstinent | 50.0 | 70.4 | 67.8 | 39.0 | 31.5 |  | 51.2 | 70.9 | 69.4 | 39.7 | 34.4 |  |
| **Abstinence from all illicit drugs or misuse of Rx, N (%)** | Abstinent | 37.6 | 24.2 | 24.6 | 46.2 | 49.2 | 0.020^C^ | 36.7 | 25.3 | 20.4 | 46.6 | 46.9 | <0.001^C^ |
|  | Non-abstinent | 62.4 | 75.8 | 75.4 | 53.8 | 50.8 |  | 63.3 | 74.7 | 79.6 | 53.4 | 53.1 |  |
| **Withdrawal (SOWS)** | Mean (SD) | 7.5 (15.9) | 11.1 (18.8) | 9.0 (16.6) | 2.6 (5.3) | 9.5 (19.5) | <0.001^R^ | 7.7 (13.8) | 9.5 (14.0) | 9.9 (16.1) | 2.9 (6.2) | 8.9 (15.8) | <0.001^R^ |
| **Depression (BDI-II)** | Mean (SD) | 7.3 (11.6) | 10.1 (11.9) | 8.4 (9.4) | 4.5 (7.8) | 7.7 (16.1) | 0.004^R^ | 7.4 (10.0) | 10.5 (12.8) | 8.7 (7.9) | 5.1 (8.2) | 6.1 (9.2) | 0.005^R^ |
| **Pain at its worst (BPI)** | Mean (SD) | 4.0 (4.0) | 5.1 (3.7) | 4.3 (3.7) | 3.4 (3.8) | 3.8 (3.9) | 0.050^R^ | 4.2 (3.5) | 5.1 (3.7) | 4.8 (3.5) | 3.6 (3.6) | 3.7 (3.3) | 0.014^R^ |
| **Physical HRQoL  (SF-12 PCS)** | Mean (SD) | 49.5 (9.7) | 49.2 (8.7) | 48.7 (8.1) | 49.1 (9.5) | 51.1 (11.9) | 0.517^R^ | 49.8 (8.3) | 48.7 (8.5) | 46.5 (7.6) | 49.1 (9.0) | 52.8 (7.2) | <0.001^R^ |
| **Physical HRQoL  (SF-12 MCS)** | Mean (SD) | 43.3 (14.8) | 40.6 (14.3) | 41.9 (13.7) | 43.1 (14.5) | 46.8 (15.5) | 0.045^R^ | 44.2 (12.6) | 40.0 (12.4) | 39.7 (11.9) | 42.9 (12.3) | 50.8 (10.4) | <0.001^R^ |
| **Probable serious mental illness  (K6, SDS), N (%)** | No probable serious mental illness | 91.5 | 91.0 | 92.7 | 92.8 | 89.2 | 0.900^C^ | 92.2 | 89.7 | 91.8 | 93.1 | 93.8 | 0.787^C^ |
|  | Probable serious mental illness | 8.5 | 9.0 | 7.3 | 7.2 | 10.8 |  | 7.8 | 10.3 | 8.2 | 6.9 | 6.3 |  |
| **Employment status, N (%)** | Employed | 47.1 | 39.0 | 43.4 | 55.1 | 46.8 | 0.405^C^ | 46.2 | 41.6 | 44.9 | 52.1 | 46.3 | 0.637^C^ |
|  | Unemployed | 52.9 | 61.0 | 56.6 | 44.9 | 53.2 |  | 53.8 | 58.4 | 55.1 | 47.9 | 53.7 |  |

Abbreviations: BDI-II, Brief Depression Index-II; BMI, body mass index; BPI, Brief Pain Inventory; BUP-XR, buprenorphine extended release; C, chi-squared based p-value; K6, Kessler’s psychological distress scale; HRQoL, health-related quality of life; m, months; R, regression-based p-value; SF-12 MCS, 12-Item Short Form Mental Component Summary; SF-12 PCS, 12-item Short Form Physical Component Summary; SOWS, Short Opiate Withdrawal Scale; SDS, Sheehan disability scale; UDS, urine drug screen.
